# Small bowel infarction due to fibro muscular dysplasia: a case report and literature review

**DOI:** 10.1186/1757-1626-3-79

**Published:** 2010-04-06

**Authors:** Sanjay Dalmia, Amir Hussain

**Affiliations:** 1 Russells Hall Hospital, Pensnett Road, Dudley, West Midlands, DY1 2HQ, UK; 2 Mission of Mercy Hospital, Park Street, Kolkata, India

## Abstract

**Introduction:**

We describe a rare case of small bowel infarction due to fibro muscular dysplasia in superior mesenteric artery in a young patient.

**Case presentation:**

A 28 year old Asian female presented with acute onset left sided abdominal pain and watery diarrhea. She had a laparotomy due to further deterioration. It showed infracted small intestine, gall bladder and parts of liver. Abdomen had to be closed without any therapeutic procedure. She died in early post operative period. Autopsy showed fibro muscular dysplasia of superior mesenteric artery.

**Conclusion:**

Fibro muscular dysplasia of SMA is rare, is treatable but has a high mortality.

## Introduction

We report a case of 28 year old female who presented with acute abdomen and subsequent laparotomy showed complete infarction of small bowel due to fibro muscular dysplasia of SMA affecting the distribution of superior mesenteric artery with occlusion of celiac axis. Fibro muscular dysplasia (FMD) is a non-atherosclerotic, non-inflammatory vascular occlusive disease that most commonly affect the renal and internal carotid arteries. On rare occasions it also affects other arteries.

## Case presentation

A 28-year-old Asian female presented to accident and emergency department with 6 weeks history of intermittent left sided abdominal pain, which got worse over last 48 hours. Abdominal pain was associated with 2 episodes of watery diarrhea not mixed with blood and several episodes of vomiting. Drug history included nothing else apart from oral contraceptive pill. On examination she was comfortable and afebrile with pulse rate of 96 and blood pressure of 160/96. Abdomen was soft but tender in left iliac fossa and left lumber region with no guarding or rebound tenderness. Digital rectal examination was unremarkable. Full blood count showed white cell count to be elevated at 24.5 × 10^6^/L. Urea, electrolyte, amylase and liver function tests were all within normal limits. Flexible sigmoidoscopic examination up to distal descending colon did not reveal any abnormality. Computer tomographic scan of the abdomen showed dilated large bowel up to splenic flexure along with dilated loops of small bowel. She was started on supportive treatment with IV Normal Saline, O2 inhalation, catheterization and antibiotics. However after treatment she failed to respond and progressively became more unwell. Gradually she developed hypotension and oliguria. Arterial blood gas analysis at 4 litre of oxygen showed compensated acidosis with following picture:

Po2-10.7 kPa

PCO2-4.04 kPa

Hydrogen ion - 43.4 nmol/litre

HCO3-17.5 nmol/litre

**Figure 1:**
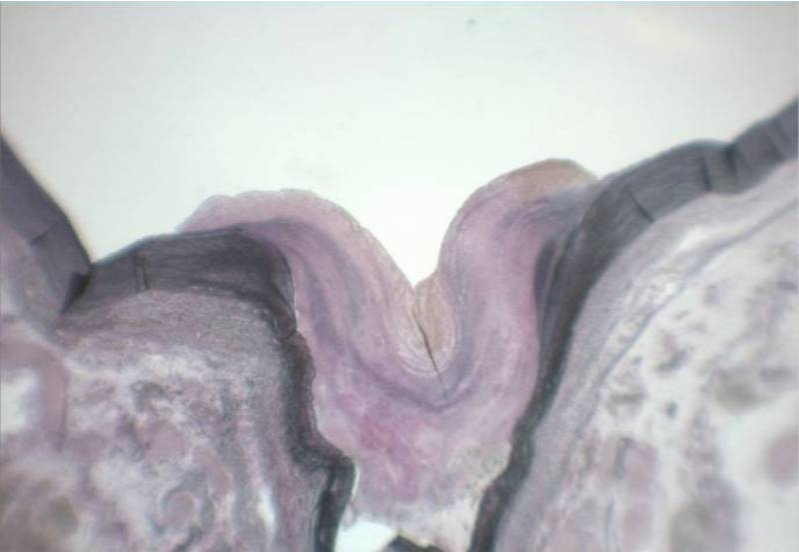
**×10-Elastica van Gieson stain - Superior mesenteric artery origin**.

**Figure 2:**
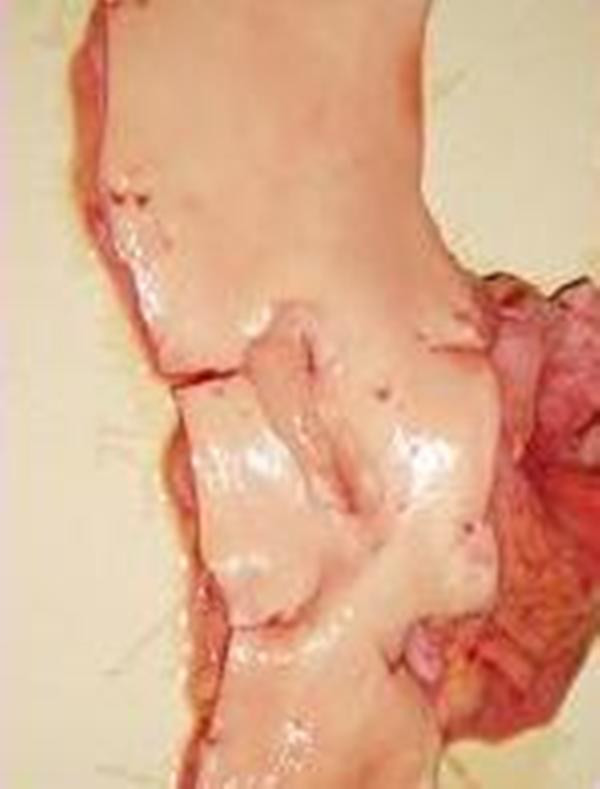
**Macroscopic picture showing origin of coeliac trunk and superior mesenteric artery**.

On reassessment of abdomen it was more tender with both guarding and rebound tenderness. She underwent laparotomy which showed complete infarction of small bowel, Gall bladder and spleen. Large bowel was infracted up to splenic flexure. Liver also appeared ischaemic. No procedure could be carried out and abdomen was closed. She subsequently died after around six hours in the post operative period. An autopsy was requested in view of operative findings. Post mortem examination confirmed the presence of organized thrombus at the origin of celiac and superior mesenteric arteries. In the aorta there was eccentric intimal thickening with loss of smooth muscle and a proliferation of elastic tissue [Figure 1]. The nature of the lesion was confirmed as intimal fibro muscular dysplasia and thrombosis causing stenosis and subsequent occlusion of the origin of superior mesenteric and celiac artery [Figure 2]. Intimal fibro muscular dysplasia of aorta causing stenosis of the origin of superior mesenteric and celiac artery was rare in medical literature.

## Discussion

FMD is an idiopathic non-atherosclerotic and non-inflammatory vascular disease that primarily affects medium and small size arteries. It is more commonly seen in female patients (M: F ratio 1:3.5) in the middle thirds of their lives [1]. It causes arterial lumen to become irregular, so thrombus formation and subsequent distal embolisation can occur. Most commonly involved sites include renal (63%-89%) and cerebral arteries (25%-38%). Less commonly affected sites are mesenteric (9%) (Table 1), subclavian (9%) and iliac (5%) arteries [2,3]. Multi vessel disease occurs in 24%-28% of patients. Diagnosis of mesenteric FMD is challenging and it can closely mimic vasculitis and atherosclerosis. Only medial fibroplasia gives a characteristic string of beads appearance on angiogram, rest of the types may be indistinguishable from atherosclerosis [3]. Occurrence of FMD in variety of vessels is responsible for wide range of symptoms including hypertension (Renal), stroke (carotid), headache (cerebral), abdominal pain (mesenteric) and claudication (iliac/femoral) [3,4]. Although a variety of genetic, mechanical and hormonal factors have been proposed, the cause of FMD remains unknown. Cigarette smoking and hypertension are associated with increased risk of this condition [5]. No association has been found between FMD and use of oral contraceptives or abnormalities of endogenous sex hormones [5]. Genetic factors may play a role in the development of FMD as the disease is more common in the first degree relatives of patients with FMD of renal arteries [6]. Rushton [7] presented evidence consistent with a pattern of automosomal dominant inheritance with variable penetrance. Since there is evidence of a genetic preponderance, first degree relatives of patients with FMD may be screened for detection of asymptomatic disease and early intervention[7]. Harrison and McCormack [8] in 1971 established criteria for the pathological classification of FMD. Three main categories of FMD are described, based on the involvement of the arterial layer: medial, intimal and periarterial or adventitial. Medial FMD can be further characterized in to medial fibroplasia, perimedial fibroplasia, and medial hyperplasia. Medial fibroplasia is most common (60-70%) and often affecting the distal two thirds of the main artery and its branches, giving an appearance of "strings of beads" on arteriogram. Perimedial fibroplasia occurs in 15-20% of cases and medial hyperplasia is less common and account for 5% to15% of cases. Symptoms of mesenteric arterial stenosis and/or occlusion include nausea, vomiting, abdominal pain, anorexia and weight loss regardless of the cause [9]. Leadbetter and burkland [9] first described FMD in the renal artery of a small child in 1938 and FMD was thought to be restricted to the renal and carotid arteries. Palubinskas and Ripley [10] first reported a case of caeliac and mesenteric arteries in 1964. Mettinger in 1982 reviewed the literature on FMD and there were only 14 cases involving mesenteric arteries, none involving celiac axis and one splenic artery. In symptomatic patients with mesenteric FMD, revascularisation is necessary but by the time symptoms of abdominal pain, weight loss, nausea and vomiting develops, the disease has progressed to a critical stage and surgery is poorly tolerated [11].

**Table 1:** Reported cases of intestinal infarction due to fibro muscular dysplasia

Author	Pathology	Treatment offered	Result
Yamaguchi et al(Am J Gastroenterol, 1996, **91**(8):1635-8)	Stenosis of jejunal and sigmoid branch	Conservative treatment leading to stenosis	Favourable
Hamed et al.(J Ped Surg, 1997, **32**(9):1379-80)	SMA Occlusion due to intimal Fibroplasia leading to intestinal gangrene	Exploratory Laparotomy	Unfavourable-Died
Mertens et al. (Acta chir belg, 2005, **105**: 523-527)	Occlusion of SMA and celiac trunk leading to visceral ischaemia	Reimplantation of Superior Mesenteric Artery	Favourable-Well two years after surgery

## Conclusion

Fibro muscular dysplasia of SMA presents dramatically. Early diagnosis can be life saving. However outcome is generally poor in most cases.

## Consent

All reasonable attempts to gain consent have been made after patient has died the patient is anonymous there is no reason to think that the patient or their family would object to publication

## Competing interests

The authors declare that they have no competing interests.

## Authors' contributions

AH conceived the idea, performed literature search and started writing the manuscript. SD obtained results and pictures. SD further contributed to manuscript, finalized the manuscript and submitted it. All the authors read and approved the final manuscript.
